# Kinetic and modeling data on phenol removal by Iron-modified Scoria Powder (FSP) from aqueous solutions

**DOI:** 10.1016/j.dib.2018.08.068

**Published:** 2018-08-29

**Authors:** Masoud Moradi, Maryam Heydari, Mohammad Darvishmotevalli, Kamaladdin Karimyan, Vinod Kumar Gupta, Yasser Vasseghian, Hooshmand Sharafi

**Affiliations:** a Research Center for Environmental Determinants of Health, Kermanshah University of Medical Sciences, Kermanshah, Iran; b Department of Environmental Health Engineering, Faculty of Public Health, Tehran University of Medical Sciences, Tehran, Iran; c Environment Research Center, Isfahan University of Medical Sciences, Isfahan, Iran; d Environmental Health Research Center, Kurdistan University of Medical Sciences, Sanandaj, Iran; e Department of Applied Chemistry, University of Johannesburg, Johannesburg, South Africa; f Students Research Committee, Kermanshah University of Medical Sciences, Kermanshah, Iran

**Keywords:** Phenol, Iron-Modified scoria, RSM, Aqueous environment

## Abstract

Phenol present in industrial effluents is a toxicant matter which causes pollution of environments aqueous. In this work, scoria was modified by iron in order to increasing of adsorbent efficiency and effective removing of phenol. Effects of independent variables including pH, adsorbents dosage, contact time and adsorbate concentration on removing of phenol were studied by response surface methodology (RSM) based on the central composite designs (CCD). The characterization of raw scoria powder (RSP) and Iron-modified Scoria Powder (FSP) was determined via Fourier transform infrared spectroscopy (FTIR), X-ray diffraction (XRD), scanning electron microscopy (SEM) and Energy-dispersive X-ray spectroscopy (EDS). The obtained data showed modification by iron caused the growth of new crystalline of iron oxide on the surface of FSP. Evaluated data by RSM indicated the all variables especially pH are effective in removing of phenol (*P*-value < 0.001) and optimum condition was obtained at pH = 5, phenol concentration = 50 mg/l, adsorbent dosage = 1 g/l and contact time = 100 min to the value of 94.99% with desirability of 0.939. Results revealed that data were fitted by Langmuir isotherm (*R*^2^ = 0.9938) and pseudo second order kinetic (*R*^2^ = 0.9976). It was found that iron causes increasing the site active of scoria and let to significant removal of phenol.

## Specifications Table

**Table:** 

Subject area	Environmental Health Engineering
More specific subject area	Environmental Chemistry
Type of data	Tables, figures
How data was acquired	XRD, FTIR, SEM and EDS techniques were used to determine the characteristics of adsorbent. Response surface methodology (RSM) was used to analyzing of experiments data to determine the effects of independent variables and define the optimum condition. Moreover, the obtained data were fitted by isotherms and kinetics equations
Data format	Raw, analyzed
Experimental factors	All samples were kept in polyethylene bottles in a dark place at room temperature.
Experimental features	Phenol was prepared and measured according to standard methods. Scoria was modified by iron in order to removal of phenol from aqueous solution.
	The all above mentioned parameters were analyzed according to the standard method for water and wastewater treatment handbook [1].
Data source location	Kermanshah city, Iran
Data accessibility	Data are included in this article
**Related research article**	M. Moradi, A.M. Mansouri, N. Azizi, J. Amini, K. Karimi, K. Sharafi, Adsorptive removal of phenol from aqueous solutions by copper (Cu)-modified scoria powder: process modeling and kinetic evaluation, Desalin Water Treat. 57 (2016) 11820–11834. (Published).

## Value of the data

• The obtained data of this study showed that Iron modification effect on adsorbent led to increasing of equilibrium sorption capacity for removal of phenol.
• Due to cheap and high availability of this type of adsorbent in Iran, the efficiency of it can be improved by making these simple modifications and so the application of it in water and wastewater treatment will be increased.
• The obtained data of present study can be used for design and development of future similar studies. Because in this study, the optimal conditions for the removal of phenol by FSP are determined. Therefore, the range of future study variables can be determined based on the optimal conditions of this study.
• The raw data of this study was analyzed using the RSM method. Therefore, the results related to the optimization conditions and the determination of the effect of each parameter will be very understandable for other researchers.

## 1 Data

The maximum efficiency of for phenol removal was obtained at pH = 3, phenol concentration = 50 mg/l, adsorbent dosage = 1 g/l and contact time = 100 min (Table 1). Results demonstrated coefficient (*R*^2^) and *R*^2^-adj value are 0.978 and 0.975 for phenol removal that suggested proper correlations between the response and variables ( Tables 2 and 3). The optimum condition was obtained for pH = 5, phenol concentration = 50 mg/l, adsorbent dosage = 1 g/l and contact time = 100 min to the value of 94.99% with desirability of 0.939 (Table 4). The percent of error between mathematical design and experimental study is 3.81% that suggested the close value of both actual and predicted data (Table 5). Results revealed that data were fitted by Langmuir isotherm (*R*^2^ = 0.9938) and obeyed the pseudo second order kinetic (*R*^2^ = 0.9976) ( Tables 6 and 7).

**Table 1:** Experimental conditions and results of central composite design.

**Run**	**Variables**				**Responses**			
					**Phenol removal by RSP**		**Response Phenol removal by FSP**	
	**Factor 1**	**Factor 2**	**Factor 3**	**Factor 4**	**Actual**	**Predicted**	**Actual**	**Predicted**
	**A: pumice dosage (gr/l)**	**B: Contact time (min)**	**C: pH**	**D: Phenol concentration (mg/l)**	**%**	**%**	**%**	**%**
1	1	20	11	50	19.31	18.97	31.4	29.67
2	0.2	20	11	50	6.21	6.9	13.2	13.98
3	1	20	3	50	79.68	81.46	92.6	93.95
4	0.6	80	7	150	70.52	67.65	82.14	76.37
5	1	100	11	50	29.32	27.89	35.6	33.11
6	0.6	60	7	150	65.76	65.42	72.65	75.34
7	1	100	3	250	68.61	65.04	79.26	77.06
8	0.6	60	7	150	65.76	65.42	73.71	75.34
9	0.6	60	7	100	66.27	69.58	78.65	79.19
10	0.2	100	3	250	49.84	52.97	61.96	65.16
11	0.6	40	7	150	58.57	63.19	67.45	72.57
12	0.6	60	7	150	65.76	65.42	75.64	75.34
13	0.6	60	7	200	60.73	61.26	72.33	71.14
14	0.6	60	7	150	65.76	65.42	75.64	75.34
15	0.6	60	7	150	65.76	65.42	75.64	75.34
16	0.2	100	11	50	14.17	15.82	13.2	17.04
17	0.6	60	9	150	53.06	48.87	60.28	57.67
18	1	100	3	50	89.14	90.38	100	103.81
19	0.2	20	11	250	3.94	− 1.03	10.87	8.52
20	0.4	60	7	150	57.44	59.23	65.49	69.6
21	0.8	60	7	150	69.07	65.26	81.26	76.5
22	0.6	60	5	150	73.6	75.76	84.59	86.56
23	0.2	100	11	250	8.53	7.89	16.65	13.87
24	1	100	11	250	15.19	19.96	22.36	25.76
25	1	20	3	250	59.04	56.12	67.29	64.91
26	1	20	11	250	8.93	11.04	17.48	20.03
27	0.2	20	3	250	41.01	44.05	52.32	53.39
28	0.2	20	3	50	70.38	69.38	80.2	78.26
29	0.2	100	3	50	81.34	78.31	91.7	87.73
30	0.6	60	7	150	65.76	65.42	76.33	75.34

**Table 2:** Estimated regression coefficients and corresponding to ANOVA results from the data of central composite design experiments before elimination of insignificant model terms: (FSP).

**MT**	**CE**	**SE**	**SS**	**DF**	**MS**	**FV**	**PV**	**S/NS**
Quadratic model	–	–	21,092.82	14	1506.63	95.66	< 0.0001	Significant
*A*	75.34	1.10	784.53	1	784.53	49.81	< 0.0001	Significant
B	6.90	0.98	238.37	1	238.37	15.13	0.0014	Significant
*C*	3.80	0.98	13,773.74	1	13,773.74	874.52	< 0.0001	Significant
*D*	− 28.89	0.98	1069.97	1	1069.97	67.93	< 0.0001	Significant
*AB*	− 8.05	0.98	0.15	1	0.15	9.29E-03	0.9245	Not significant
*AC*	0.096	0.99	1.56E-04	1	1.56E-04	9.92E-06	0.9975	Not significant
*AD*	− 3.125E-003	0.99	17.45	1	17.45	1.11	0.3092	Not significant
*BC*	− 1.04	0.99	41.12	1	41.12	2.61	0.1270	Not significant
*BD*	− 1.60	0.99	5.26	1	5.26	0.33	0.5721	Not significant
*CD*	0.57	0.99	3.77E + 02	1	3.77E + 02	2.39E + 01	0.0002	Significant
*A*^2^	4.85	0.99	13.92	1	13.92	0.88	0.3621	Not significant
*B*^2^	− 9.14	9.73	2	1	2	0.13	0.7267	Not significant
*C*^2^	− 3.46	9.73	27.71	1	27.71	1.76	0.2045	Not significant
*D*^2^	− 12.90	9.73	0.078	1	0.078	4.95E-03	0.9448	Significant

**CE:** Coefficient Estimate, **SE:** Standard Error, **MT:** Model Terms, **SS:** Sum of squares, **DE:** Degree of Freedom, **MS:** Mean square, **FV:** F-value, **PV:***P*-value, **S:** Significant, **NS:** Not significant.

**Table 3:** Analysis of variance (ANOVA) for fit of Phenol removal efficiency from central composite design after elimination of insignificant model terms: (FSP).

Model	SMT	SD	*R*^2^	Adj. *R*^2^	CV	AP	PRESS	PV	FV	PLF
Quadratic model	*A, B*, *C, D*, *CD*	3.97	0.989	0.978	7.51	33.95	1500.74	< 0.0001	95.66	*0.079*
PhenolRemoval(%)=+75.34+6.9A+3.8B−28.89C−8.05D+4.85CD										

**R**^**2**^**:** Determination Coefficient, **Adj. R**^**2**^**:** Adjusted R^2^, **AP:** Adequate Precision, **SMT:** Significant Model Terms, **SD:** Standard Deviation, **CV:** Coefficient Of Variation, **PRESS:** Predicted Residual Error Sum Of Squares, **FV:** F-value, **PV:***P*-value, **PLF:** Probability For Lack Of Fit.

**Table 4:** Numerical optimization for central composite design for phenol removal by FSP.

**Number**	**A: Scoria dosage (gr/l)**	**B: Contact time(min)**	**C: pH**	**D: Phenol concentration (mg/l)**	**Phenol removal by FSP (%)**	**Desirability**	
**Optimized Phenol removal calculated from central composite design**							
*1*	*1*	*100*	*5*	*50*	*94.9999*	*0.939*	**Selected**
2	1	100	5	52	94.9991	0.939	
3	1	100	5	50	95.0002	0.938	
4	1	100	5	55	95.0002	0.937	
5	1	100	5	59	95	0.936	
6	1	100	5	61	95.0001	0.935	
7	1	100	5	64	95.0001	0.934	
8	1	97	5	50	94.9801	0.931	
9	1	100	5	50	93.8081	0.929	
10	1	100	5	81	95.0002	0.926	
11	1	100	5	65	93.7232	0.923	
12	1	100	4	91	95.0002	0.922	
13	1	100	4	98	95.0002	0.92	
14	1	100	4	100	95.0002	0.919	
15	1	100	4	105	95.0001	0.917	
16	1	100	4	106	95.0002	0.917	
17	1	100	3	114	95.0002	0.916	
18	1	100	3	115	95.0002	0.916	
19	1	100	5	82	94.0105	0.913	
20	1	100	3	126	95	0.902

**Table 5:** Confirmation between optimized phenol removals calculated from mathematical design and experimental study.

**A: Scoria dosage (gr/l)**	**B: Contact time(min)**	**C: pH**	**D: Phenol concentration (mg/l)**	**Phenol removal by FSP (%)**
**Optimized phenol removal calculated from central composite design (predicted value**)				
1	100	3	50	103.81

Confirmation study of optimized Phenol removal (experimental value)				
1	100	3	50	100
	
$Error\;(\%)=\frac{Actual\;value-predicted\;value}{Actual\;\;value}\times 100$				3.81%

**Table 6:** Isotherm equation parameters for phenol adsorption on FSP.

**Adsorbent**	**Langmuir isotherm**	
FSP	*q_m_* (mg/g)	43.06
	*b*	0.11
	*R*^2^	0.9938
	**Freundlich isotherm**	
FSP	*n_T_*	5.68
	*K_f_* (mg/g(l/mg)^1/*n*^)	17.44
	*R*^2^	0.9315

**Table 7:** Kinetic model parameters for the adsorption phenol at different concentration on FSP.

**Kinetic model parameters**	**Kinetic parameters**	**FSP**
Pseudo-first-order	*K*_1_	0.1922
	*R*^2^	0.9177
Pseudo-second-order	*K*_1_	0.00487
	*R*^2^	0.9976
Pore diffusion	*K*_i_	0.9336
	*R*^2^	0.8766
Elovich	*A*	0.279
	*B*	2.75
	*R*^2^	0.9625

**Fig. 1:**
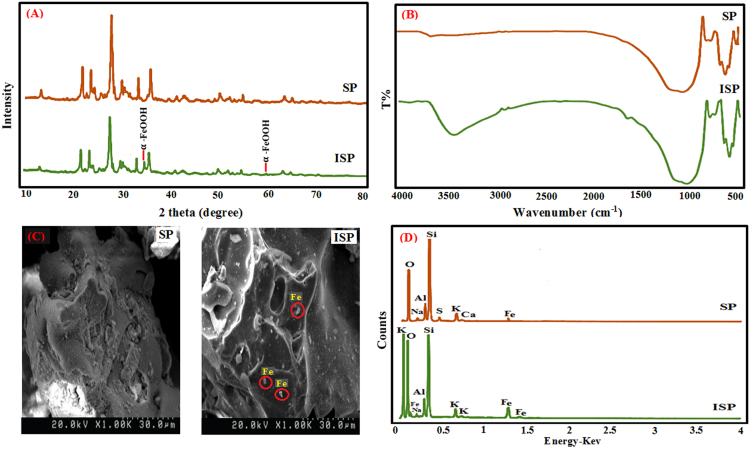
XRD patterns (A), Fourier transform infrared spectroscopy (FTIR) (B), SEM images (C) and EDS analysis of SP and FSP (D).

**Fig. 2:**
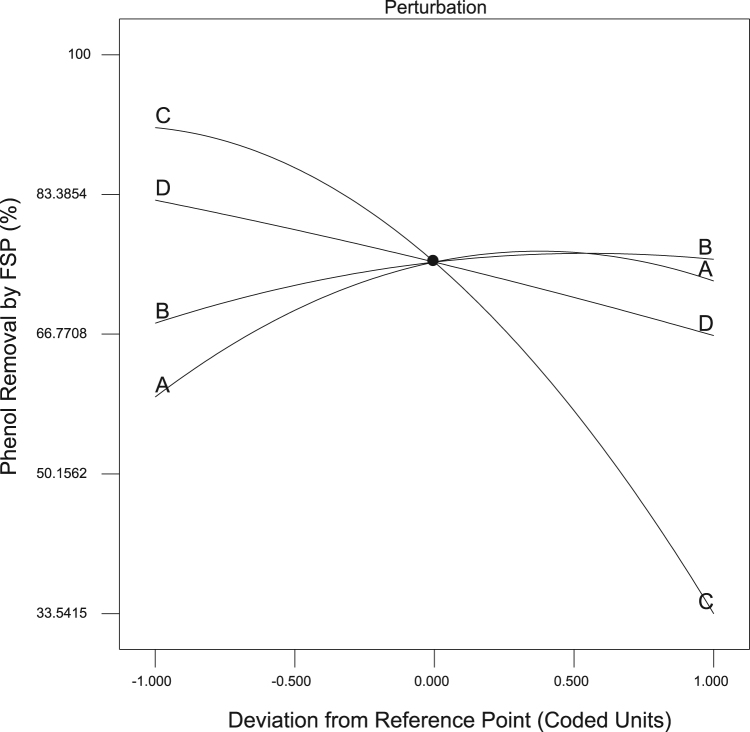
Trend of phenol removal efficiency with respect to scoria dosage (A), contact time (B), pH (C), and phenol concentration (D).

**Fig. 3:**
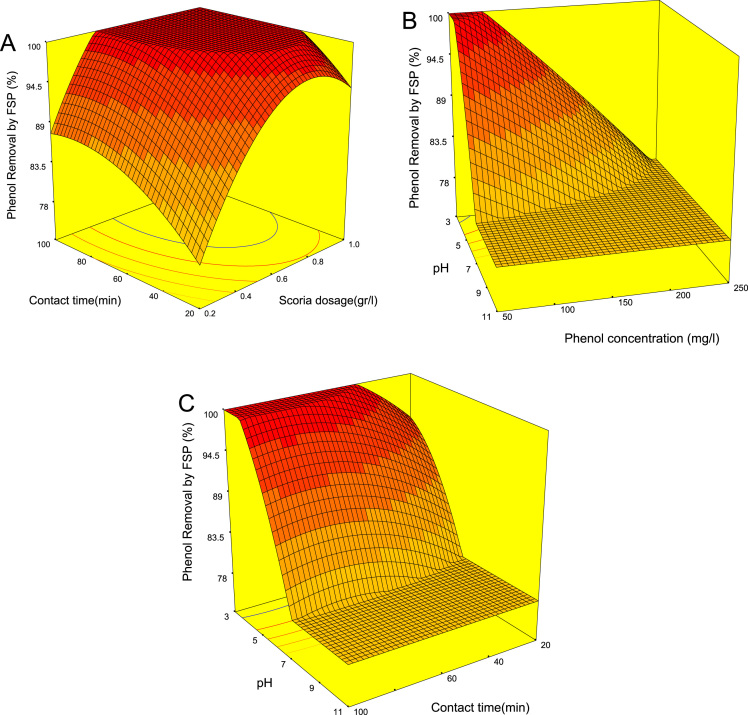
Response surface plots for phenol removal efficiency with respect to contact time and scoria dosage (A), pH and phenol concentration (B), pH and contact time (C).

**Fig. 4:**
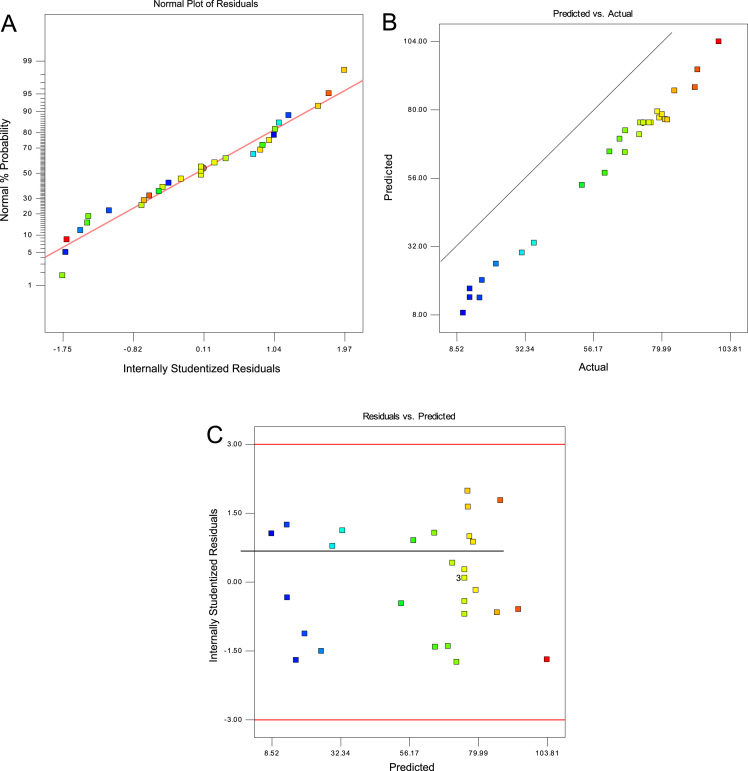
Normal probability plot of residual (A), predicted vs. actual values plot (B), and plot of residual vs. predicted response (C).

Fig. 1 showed the XRD patterns, Fourier transform infrared spectroscopy (FTIR), SEM images and EDS analysis of RSP and FSP. Trend of phenol removal efficiency with respect to scoria dosage, contact time, pH, and phenol concentration was showed in Fig. 2. The response surface plots for phenol removal efficiency with respect to scoria dosage, pH, phenol concentration, and contact time were showed in Fig. 3. In addition, Normal probability plot of residual, predicted vs. actual values plot, and plot of residual vs. predicted response were showed by Fig. 4.

## 2 Experimental design, materials and methods

### 2.1 Pumice preparation and its modification using iron

Early preparations of raw scoria powder (RSP) were performed according to Moradi et al. [15] study [2]. The raw scoria powder (RSP) was kept in Fe(NO_3_) 3.9H_2_O (0.5 m) solution at pH = 12 and 25 °C (room temperature) for 72 h, and dried at 110° C for 14 h. Not doped iron was removed via washing of modified scoria by distilled water, afterwards, FSP dried at 105 °C for 14 h [2], [3], [4].

### 2.2 Characteristics of SP and FSP

The functional groups of adsorbents were determined by Fourier transform infrared spectroscopy (FTIR) (WQF-510 Model), X-ray diffraction (XRD) model Shimadzu XRD-6000 were used for study of chemical characteristics and surface morphology of adsorbent. Scanning electron microscope (SEM) model Philips XL30 was used to evaluation the sample׳s surface topography and composition. Energy Dispersive X-Ray Spectroscopy (EDS) model EM-30AX Plus was applied for determination of chemical characterization and elemental analysis of adsorbents [5], [6].

### 2.3 Experimental design by response surface methodology (RSM)

Design of experiments (DOE) software was used to design of experiments (the required sample size). Table 8 illustrated-the experimental range and level of the independent variables. The RSM based on central composite design (CCD) as statistical tool was used to minimization of experiments number. On the other hand, optimum condition was determined through consideration of relationship between the measured responses (phenol removal) and number of independent variables [7], [8], [9], [10].

**Table 8:** Experimental range and level of the independent variables.

**Variables**	Range and level				
	− **α(**− **1.5)**	− **1**	**0**	**1**	**+ α(1.5)**
Contact Time, min	20	40	60	80	100
Adsorbent Dosage, gr/l	0.2	0.4	0.6	0.8	1
pH	3	5	7	9	11
Phenol concentration (mg/l)	50	100	150	200	250

### 2.4 Samples preparation and batch sorption studies

Phenol with molecular formula C_6_H_5_OH and molecular weight of 94.11 g/mol was purchased from the Merck Company-Germany (CAS. 108-92-5). Different concentrations of phenol (50, 100, 150, 200 and 250 mg/l) were prepared from phenol stoke (1000 mg/l). The phenol adsorption by FSP was conducted under following conditions: adsorbent dose (0.1–1 g/l), pH (3, 5, 7, 9 and 11), contacted time (20, 40, 60, 80 and 100 min) and room temperature (25 °C). The residual phenol was determined by UV/VIS spectrophotometer (Hitachi Model 100-40) at λ_max_ 500 nm [3], [11], [12].

### 2.5 The study of adsorption isotherms

Langmuir and Freundlich isotherms are the main mathematical equations for description of reaction between adsorbents adsorbate. The equilibrium adsorption capacity by adsorbent was calculated as follows [13], [14], [15], [16]:

(1) $$q_{e}=\frac{(C_{0}-C_{e})V}{m}$$

where,

*q_e_* (mg/g) is the equilibrium adsorption capacity
*C_0_* and *C_e_* are the initial and equilibrium concentration of phenol (mg/l)
*V* is the volume of solution (l)
*M* is the weight of adsorbent (g).

#### 2.5.1 Langmuir isotherm

The Langmuir isotherm is used to describe the monolayer adsorption of adsorbate on the adsorbent surface. This isotherm assumed the uniform number of adsorption sites. The nonlinear equation of Langmuir was depicted (Eq. (2)). Several equations related to Langmuir isotherm were derived from nonlinear equation (Eqs. (3), (4), (5)) [15], [16], [17].

(2) $$q_{e}=\frac{(q_{m}bC_{e})}{1+bC_{e}}$$

(3) $$\frac{C_{e}}{q_{e}}=\frac{1}{bq_{m}}+\frac{C_{e}}{q_{m}}$$

(4) $$\frac{1}{q_{e}}=\frac{1}{bq_{m}C_{e}}+\frac{1}{q_{m}}$$

(5) $$\frac{q_{e}}{C_{e}}=bq_{m}-bq_{e}$$

#### 2.5.2 Freundlich isotherm

The Freundlich isotherm assumed the multi-layer adsorption on heterogeneous adsorbent sites with unequal and non-uniform energies. The nonlinear and linear equations are presented as follow respectively [18], [19], [20], [21], [22], [23]:

(6) $$q_{e}=K_{F}{(C_{e})}^{\frac{1}{n}}$$

(7) $${\ln q}_{e}=\ln K_{F}+n^{-1}\ln C_{e}$$

### 2.6 The study of adsorption kinetics

The reaction kinetics was used to study of the factors affecting the reaction rate. The kinetics equations of pseudo-first-order (Eq. (8)), pseudo-second-order (Eq. (9)), intraparticle diffusion (Eq. (10)) and Elovich (Eq. (11)) were presented as follow:

(8) $$\ln\left(q_{e}-q_{t}\right)=\ln q_{e}-k_{1}t$$

(9) $$\frac{1}{q_{t}}=\frac{1}{q_{e}}+k_{2}t$$

(10) $$q_{t}=k_{p}t^{0.5}$$

(11) $$q_{t}=\beta \ln\left(\alpha \beta \right)+\beta \mathrm{lnt}$$
